# An in vivo study of the ameliorative effect of supplementation with *Lacticaseibacillus paracasei* Glory LP16 in immunocompromised mice

**DOI:** 10.1038/s41598-025-97430-4

**Published:** 2025-04-29

**Authors:** Weiwei Ma, Yanan Wu, Hang Sun, Yiyang Zhao, Lili Huang

**Affiliations:** College of Pharmacy, Heilongjiang University of Chinese Medicine, Harbin, 150030 Heilongjiang China

**Keywords:** *Lacticaseibacillus paracasei* Glory LP16, Cyclophosphamide, Immunomodulation, Microbial communities, Biochemistry

## Abstract

Probiotics play a beneficial role in improving the intestinal microecological balance and improving the health level and state of the host. In this study, cyclophosphamide was used to establish an immunocompromised mouse model. In the experiment, sixty mice were randomly divided into 5 groups: normal control, model control, Glory LP16 low-dose control (2 × 10^6^ CFU/mouse, LP), Glory LP16 medium-dose control (2 × 10^7^ CFU/mouse, MP), and Glory LP16 high-dose control (2 × 10^8^ CFU/mouse, HP). The mice were tested for body weight, immune organ indexes, cellular immunity indexes, humoral immunity indexes, non-specific immunity indexes, colonic histopathological notices, intestinal flora, and short-chain fatty acids. The results showed that compared with the model control, the high-dose control showed an increase in body mass gain, thymus index, spleen index, optical density index, foot-plantar thickness, number of hemolyzed vacuoles, number of antibody accumulation, NK cell activity, carbon profile phagocytosis index, macrophage neutral red phagocytosis rate, macrophage phagocytosis index, the morphology of colon tissue tended to be more like that of the normal tissue, the regulation of the intestinal bacterial flora imbalance, and an increase in short-chain fatty acids of the intestine. It is hypothesized that *Lacticaseibacillus paracasei* Glory LP16 has the function of enhancing immunity in mice.

## Introduction

The gut is a complicated and changing place that has developed special immune cells over time because it has been exposed to many harmful things^1^. Various intestinal immune cells play an important role in host immune function, thereby suppressing the onset of infection and modulating immune tolerance to communal bacteria and ingested antigens^2,3^. Special immune cells have been developed over time because the gut has been exposed to many harmful things^4^. These interactions can help host organisms fight against illnesses like inflammation and infectious diseases by making their immune systems stronger^5^. Thus, the immunity of the organism can be improved by modulating the gut microflora^6^.

The Food and Agriculture Organization of the United Nations/World Health Organization (FAO/WHO) has given a definition of a probiotic, which is "a living microorganism that, when administered in sufficient quantities, provides a health benefit to the host"^7,8^. Although the health benefits of probiotics are well known, there is still a need to understand the underlying mechanisms by which they interact with immune cells to stimulate immunomodulatory effects^9^. At present, the size of the global probiotics market is developing rapidly, and the function of probiotics in enhancing immunity has been widely acknowledged^8^. With the support of China’s Ministry of Science and Technology, the relationship between probiotics and human health has attracted the attention of the academic community, indicating that China’s probiotics market has a bright future^8,10^.

Because *Lacticaseibacillus* strains can create bioactive substances that lower the prevalence of different human diseases, they are regarded as safe and positive probiotics for use in the production of “natural food” products^11^. *Lacticaseibacillus paracasei* is a Gram-positive homofermentative lactic acid bacterium of the Lactobacillus species, which is commonly found in the human intestinal tract, oral cavity, and is commonly used in the field of dairy fermentation. *Lacticaseibacillus paracasei* can reduce inflammation, treat allergies, inhibit cancer and fight proliferation. *Lacticaseibacillus paracasei* can also improve the immunity of the host organism. This has become a hotspot of research because of its probiotic properties^12^. Kang XN found that four inflammatory factors IL-1β, IL-6, IL-8, and TNF-α decreased significantly after treatment of animal enteritis model using *Lacticaseibacillus paracasei* GZ0613^13^. It proved that *Lacticaseibacillus paracasei* GZ0613 has obvious preventive and therapeutic effects on animal enteritis^13^. Huang found that *Lacticaseibacillus paracasei* R3 could improve the balance of the host immune system by regulating DSS-induced colitis in mice by regulating the Treg /Th17 cell balance in DSS-induced colitis in mice, which significantly improved the symptoms and pathological damage in colitis mice and affected the immune function^14^. It has been confirmed that probiotics can regulate immunity and inhibit intestinal pathogenic microorganisms. *Lacticaseibacillus paracasei* has been confirmed to produce health benefits to the host through a variety of ways, but there are few studies on the regulation of *Lacticaseibacillus paracasei* on host immunity. Moreover, the effects of different types of *Lacticaseibacillus paracasei* may be different, and there are few studies on *Lacticaseibacillus paracasei* Glory LP16. Therefore, it is of great significance to explore how *Lacticaseibacillus paracasei* Glory LP16 regulates intestinal microorganisms to improve immunity^15^.

Cyclophosphamide (CTX) is an immunosuppressant that can inhibit the humoral and cellular immune responses of animals and so on. It is commonly used in the establishment of immunosuppression models, and is a broad-spectrum antitumor drug commonly used in the clinic^16,17^.

In this study, *Lacticaseibacillus paracasei* Glory LP16 was selected to perfuse mice and an immunocompromised mouse model was established with CTX according to the methods of the Technical Code for the Inspection and Evaluation of Health Foods^18^. The effects on body weight, thymus and spleen indices, cellular immune function, humoral immunity, non-specific immunity, colonic tissue morphology, intestinal flora composition and short-chain fatty acid content of the mice were measured. The immunomodulatory function of *Lacticaseibacillus paracasei* Glory LP16 was evaluated to provide a theoretical basis for the rational application of probiotic micro-ecological preparations and the development of health foods.

## Results

### Effect of Glory LP16 on body weight in mice

Compared with the initial body weight, MC, LP, MP and HP showed some growth. It proved that the mouse organism itself had some recovery ability, but it was still significantly lower than that of the control group (*p* < 0.05). Compared with MC, LP, MP and HP mice showed a highly significant increase in body weight gain (*p* < 0.01) (Table 1). This indicated that gavage of all doses of Glory LP16 was effective in body weight gain in immunocompromised mice.

**Table 1:** Effect of Glory LP16 on body weight of mice.

Groups	Initial weight (g)	Ultimate weight (g)
NC	20.01 ± 0.3	28.50 ± 0.2^a^
MC	20.06 ± 0.4	23.24 ± 0.3^e^
LC	20.07 ± 0.4	24.53 ± 0.5^d^
MC	20.06 ± 0.3	26.79 ± 0.7^c^
HC	20.12 ± 0.3	27.45 ± 0.4^b^

Different lowercase letters indicate significant differences (*p* < 0.05), n = 12.

### Effect of Glory LP16 on immune organ indices in mice

Thymus and spleen are very important immune organs of the organism, so the level of thymus index and spleen index can indirectly reflect the overall immune level of the organism, which is of great significance in the evaluation of immune function^19^. As shown in Table 2, compared with NC, MC thymic and splenic indices were reduced, with highly significant differences (*p* < 0.01). Compared with the model control, the thymus and spleen indices of mice in the MP and HP group were highly significant (*p* < 0.01), while there was no significant difference in the thymus and spleen indices in the LP group compared with them (*p* > 0.05). The results indicated that the gavage of high doses significantly increased the thymic index and splenic index of mice, promoted the recovery and development of immune organs in immunocompromised mice, and resisted the effects of immunosuppression on the development of immune organs.

**Table 2:** Effect of Glory LP16 on thymus and spleen index in mice.

Groups	Thymus index	Splenic index
NC	2.56 ± 0.09^a^	5.47 ± 0.13^a^
MC	1.79 ± 0.10^e^	4.37 ± 0.14^e^
LC	1.93 ± 0.14^d^	4.63 ± 0.14^d^
MC	2.15 ± 0.12^c^	5.06 ± 0.12^c^
HC	2.34 ± 0.16^b^	5.22 ± 0.09^b^

Different lowercase letters indicate significant differences. (*p* < 0.05), n = 12.

### Effect of Glory LP16 on cellular immune function in mice

**Fig. 1:**
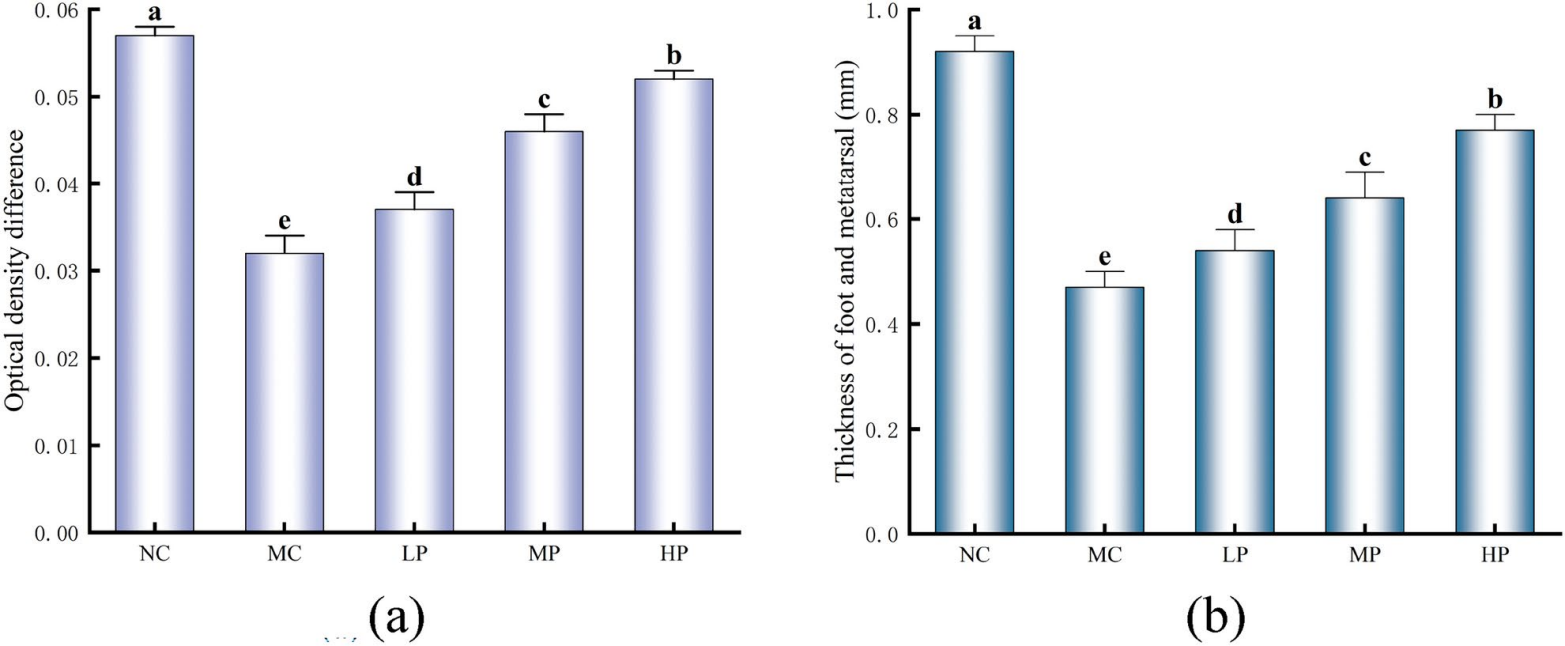
Effect of Glory LP16 on cellular immune function in mice. (**a**) Difference in optical density; (**b**) Thickness of the metatarsal region of the foot. Note: Different lowercase letters indicate significant differences (*p* < 0.05), n = 12.

The results of the Con A-induced mouse splenic lymphocyte transformation assay are shown in Fig. 1a, which shows a significant decrease in MC optical density difference compared to NC (*p* < 0.01). This indicates that CTX can lead to a decrease in splenic lymphocyte activity, which in turn affects cellular immunity. Compared with MC, LP, MP, and HP were all highly significant (*p* < 0.01). The results suggest that administration of different doses of Glory LP16 to mice can stimulate splenic lymphocytes to different degrees, increase cell activity and promote their proliferation to enhance immune function. As can be seen in Fig. 1b, the foot-plantar thickness was significantly decreased in the model group compared with the control group, with a highly significant difference (*p* < 0.01). Compared with MC, LP, MP and HP foot-plantar thickness increased significantly, with highly significant difference (*p* < 0.01). The results indicated that the gavage of Glory LP16 could enhance the delayed metamorphosis reaction in mice to a certain extent.

### Effect of Glory LP16 on humoral immune function in mice

**Fig. 2:**
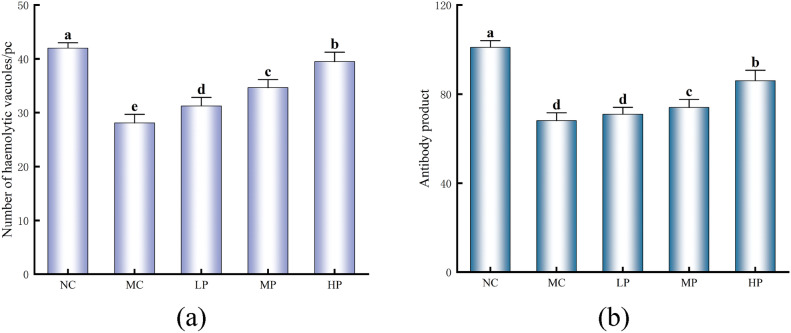
Effect of Glory LP16 on humoral immune function in mice. (**a**) Number of hemolyzed empty spots; (**b**) Antibody product. Note: Different lowercase letters indicate significant differences (*p* < 0.05), n = 12.

As shown in Fig. 2a, the difference in the number of hemolytic vacuoles was highly significant (*p* < 0.01) in MC compared to NC. The differences in LP, MP and HP were also highly significant (*p* < 0.01) compared to MC. As shown in Fig. 2b, the difference in the number of antibodies accumulated in the MC group was highly significant (*p* < 0.01) compared to the NC group. Compared with MC, the number of MP and HP antibody accumulation was significantly higher (*p* < 0.05), in which the number of HP antibody accumulation was significantly higher than that of MC, and the difference was highly significant (*p* < 0.01). The results indicated that Glory LP16 could enhance the humoral immune function of mice to a certain extent.

### Effect of Glory LP16 on non-specific immunity in mice

**Fig. 3:**
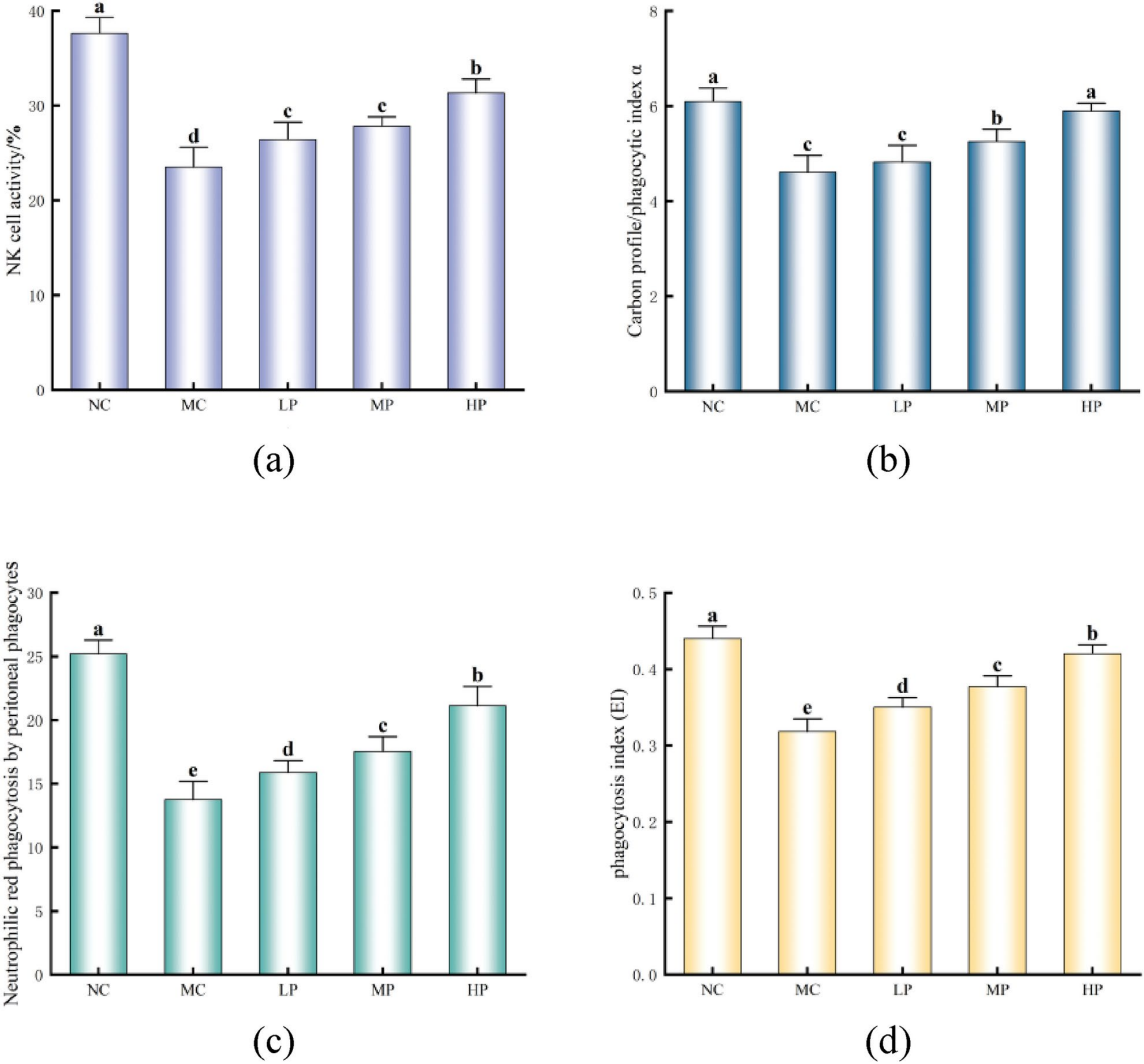
Effect of Glory LP16 on non-specific immunity in mice. (**a**) NK-cell activity; (**b**) Carbon profile/phagocytosis index α; (**c**) Neutrophilic red phagocytosis by peritoneal phagocytes; (**d**) Phagocytosis index (EI). Note: Different lowercase letters indicate significant differences (*p* < 0.05), n = 12.

As shown in Fig. 3a, the NK cell activity of MC was highly significantly reduced compared with NC (*p* < 0.01); the NK cell activity of LP, MP, and HP was highly significantly enhanced compared with MC (*p* < 0.01); and the difference was not significant for MP compared with LP (*p* > 0.05). As shown in Fig. 3b, the carbon profile phagocytosis index of MC was highly significantly reduced (*p* < 0.01) compared with NC; the carbon profile phagocytosis index of MP and HP was highly significantly increased (*p* < 0.01) compared with MC while the difference of LP was not significant (*p* > 0.05). As can be seen from Fig. 3c, macrophage neutral red phagocytosis was highly significantly reduced in MC compared with NC (*p* < 0.01); LP, MP, and HP macrophage neutral red phagocytosis was highly significantly increased in LP, MP, and HP compared with MC (*p* < 0.01). From Fig. 3d, it can be seen that the macrophage phagocytic index α of MC was extremely significantly reduced (*p* < 0.01) compared with NC; and the macrophage phagocytic index α of LP, MP, and HP was extremely significantly increased (*p* < 0.01) compared with MC. From the above results, it can be shown that Glory LP16 can improve the non-specific immunity of mice to a certain extent.

### Effects of different doses of Glory LP16 on colon tissue in mice

**Fig. 4:**
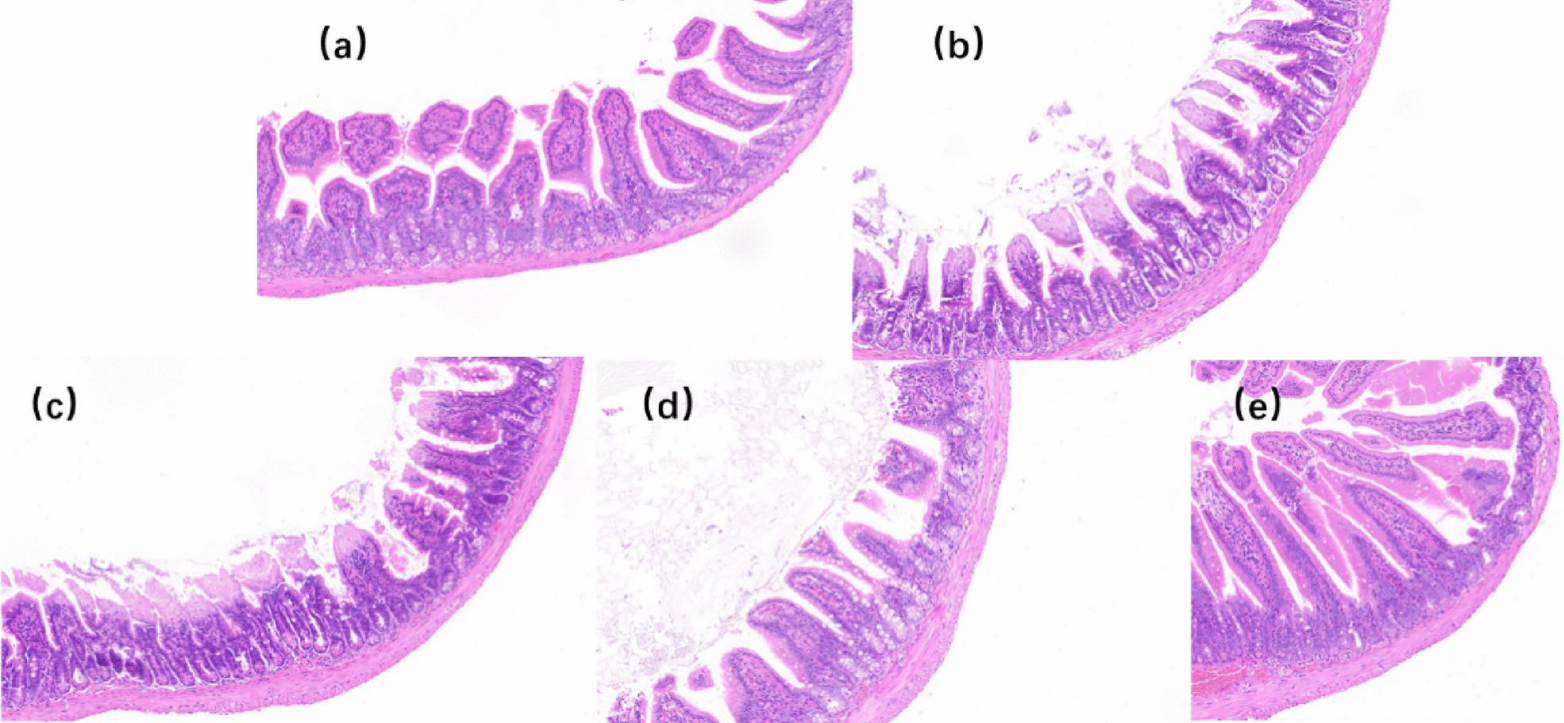
Effects of different doses of Glory LP16 on colon tissue in mice. (**a**) Staining results of colon HE in normal mice; (**b**) Staining results of colon HE in the model group; (**c**) The results of colon HE staining in the low-dose group were obtained; (**d**) Staining results of colon HE in mice in the middle-dose group; (**e**) Staining results of colon HE in mice in the high-dose group. Note: Different lowercase letters indicate significant differences (*p* < 0.05), n = 12.

From Fig. 4, the effect of different doses of Glory LP16 on the colon tissue of mice in the blank group was normal, the epithelial cells of the mucosal layer were neatly arranged and tight, and there was no inflammatory cell infiltration. In the model control, the colon tissue structure was abnormal, the goblet cells were reduced, and a large number of inflammatory cells were infiltrated. With the increase of Glory LP16 dose, the colon tissue morphology tends to normal tissue, goblet cells gradually increase, and the degree of inflammatory cell infiltration decreases.

### Effects of Glory LP16 on intestinal flora in mice

**Fig. 5:**
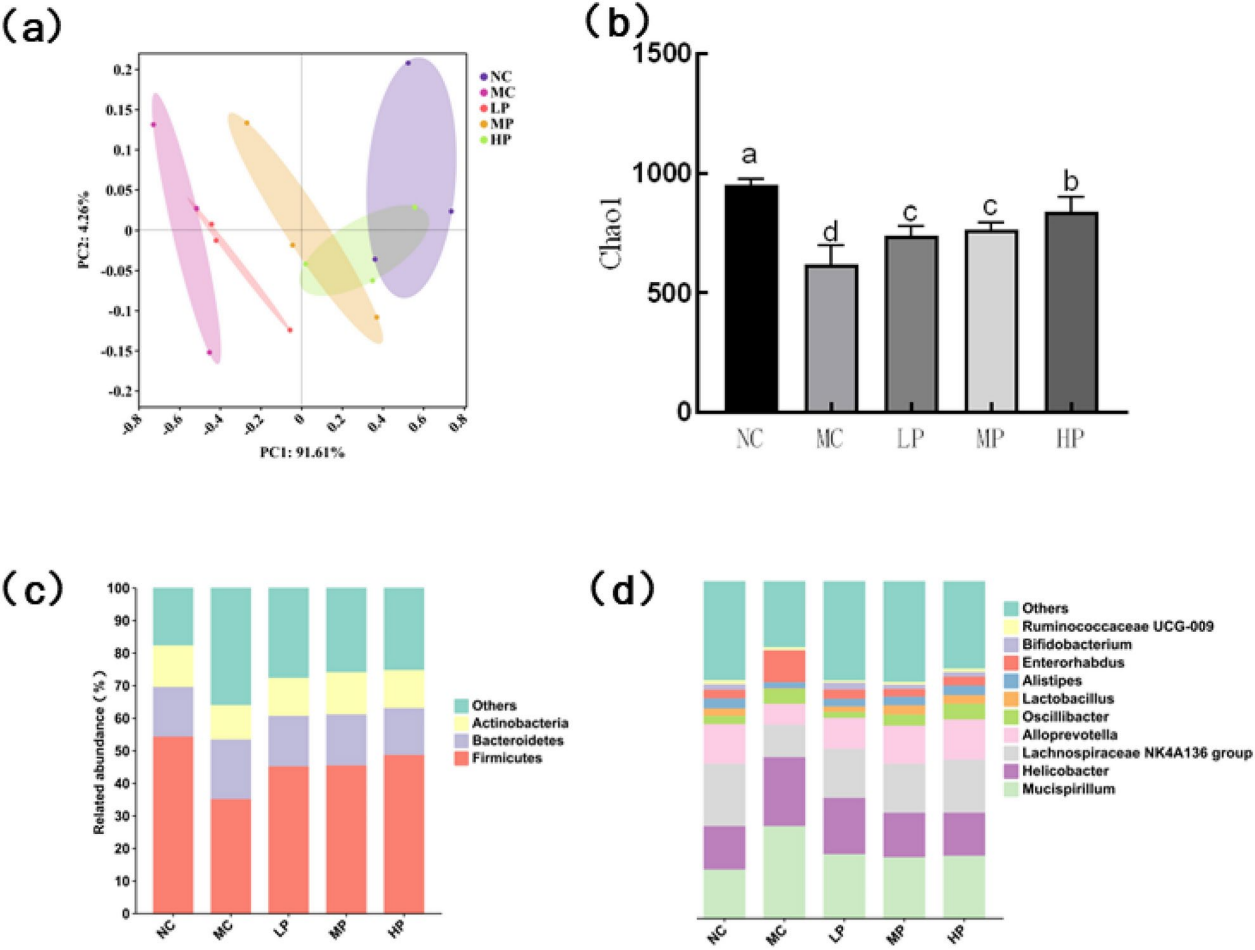
Effects of Bacillus para-casei Glory LP16 on intestinal flora in mice. (**a**) PCA of each group; (**b**) α diversity analysis; (**c**) Intestinal flora structure at phylum level; (**d**) Intestinal flora structure at Genus level. Note: Different lowercase letters indicate significant differences (*p* < 0.05), n = 12.

By sequencing 16S rDNA of mouse intestinal contents, it can be seen from the results of principal component analysis in Fig. 5a that there is a significant deviation between the MC group and the NC group, while the NC group and the HP group are closer. The α diversity analysis shows that, compared with the NC group (Fig. 5b**)**, the Chao1 index of the MC group is significantly decreased (*p* < 0.01). After intervention, the Chao1 index of each dose group was higher than that of MC group, and the Chao1 index was increased with the increase of Glory LP16 dose. At the gate level (Fig. 5c), *Firmicutes*, *Bacteroidetes* and *Actinobacteria* were predominant in different treatment groups. Compared with the control group, significant changes were found in the intestinal microbial structure of mice in the model group, mainly the decline in the abundance of *Firmicutes* and *Actinobacteria*. The abundance of intestinal *Firmicutes* increased after the treatment of Glory LP16 compared with the model group. At the genus level (Fig. 5d), it was observed that *Enterorhabdus*, *Oscillibacter*, *Helicobacter* and *Mucispirillum* increased in immunoweakened mice compared with the NC group. *Bifidobacterium*, *Alistipes, Lactobalillus*, *Alloprevotella*, *Lachnospiraceae NK4A136 group* decreased. The relative levels of *Helicobacter*, *Mucispirillum* and other bacteria were decreased after the intervention of Glory LP16. The relative levels of Bifidobacterium, *Lachnospiraceae NK4A136 group* and *Alloprevotella* were increased to improve intestinal balance.

### Effects of Glory LP16 on intestinal SCFAs content in mice

**Fig. 6:**
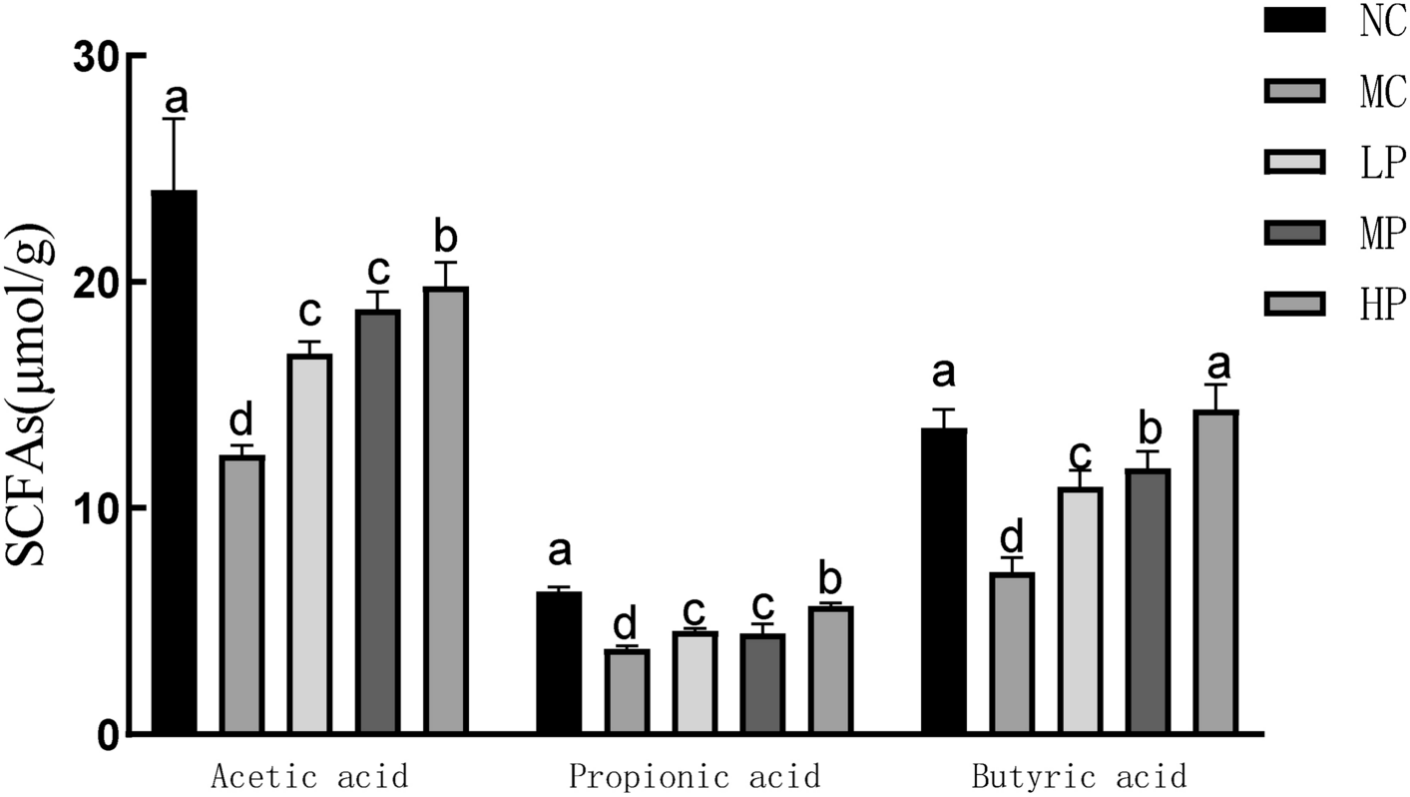
Content of short-chain fatty acids in mouse intestinal contents. Note: Different lowercase letters indicate significant differences (*p* < 0.05), n = 12.

As a very important metabolite of intestinal flora, short-chain fatty acids are closely related to immunity^20^. As shown in Fig. 6**,** compared with the blank group, the contents of acetic acid, propionic acid and butyric acid in the intestinal short-chain fatty acids of mice after CTX intervention were significantly reduced (*p* < 0.01). The contents of acetic acid, propionic acid and butyric acid in the intestinal short-chain fatty acids of mice supplemented with Glory LP16 were increased, suggesting that Glory LP16 could increase the content of short-chain fatty acids in the intestinal tract of mice, thus improving the immune ability.

## Discussion

Probiotics can regulate the balance of the intestinal microbiota and have an invaluable role in enhancing the body’s immunity^21,22^. Studies have shown that probiotics can modify the endogenous microbial or immune system activity of the host by directly or indirectly modulating the host’s gut microbiota after entry into the organism^23^. In the present study, intraperitoneal injection of CTX was able to significantly reduce the body weight of mice and their immune organ indices (thymus and spleen), inhibit their splenocyte proliferation, reduce the phagocytic activity of mouse macrophages and abnormalities in colonic tissue structure. Furthermore, the MC group exhibited an imbalance of intestinal flora and a notable reduction in short-chain fatty acid content, including acetic acid, propionic acid, and butyric acid. As a result, the immune level was significantly reduced in the MC group compared to the NC group, a data that is consistent with previous findings in the literature, and these reports also point to the inhibitory effect of CTX on immune function in mice^24,25^. The above results indicate that CTX can significantly inhibit the immune function of BALB/c mice and successfully establish a mouse immunosuppression model.

The spleen is the largest peripheral lymphatic organ in the organism and is also the site of the presence of B lymphocytes^26^. Therefore, the functional state of the spleen is essential for the maintenance of the body’s humoral immunity^27^. The thymus belongs to the central immune organs and is the site of T-lymphocyte differentiation and maturation, while T-cells play a central role in cellular immunity^27^. Therefore, the spleen and thymus can be used as one of the basic indicators of the body’s immune function, thus indirectly reflecting the body’s immune level ^28^. The thymus index and spleen index of mice were measured, and the results showed that compared with the MC group, the thymus index and spleen index of mice increased after the intervention of *Lacticaseibacillus paracasei* Glory LP16, Bu YS by gavage of Lactobacillus plantarum YRL45, the liver or spleen index and thymus index increased, which had a certain regulatory effect on the immune system^29^. The thymus and spleen, as the main immune organs of the body, play a very important role in maintaining immune homeostasis and resisting the invasion of pathogens^30^. *Lacticaseibacillus paracasei* Glory LP16 was able to restore the thymic index and spleen index of immunocompromised mice due to CTX, and further promote the development of immune organs in immunocompromised mice, thus improving the immunocompetence of immunocompromised mice.

Cellular immunity, as an important component of the body’s immune system, is mainly mediated by T lymphocytes^31^. When T-lymphocytes release lymphokines, they trigger an inflammatory response of varying degrees, and the intensity of this T-lymphocyte-mediated inflammatory response is closely related to the strength of cellular immune functions^31^. When cellular immunity is low, it may lead to incomplete antigen clearance, persistent inflammation, and even a variety of diseases^32^. In this experiment, the capacity of T lymphocytes to undergo conversion into matricellular cells for sustained value-added following stimulation by Con A, as well as the foot-plantar thickness of mice, were determined. Furthermore, Glory LP16 was observed to markedly enhance the value-added capacity of lymphocytes and the foot-plantar thickness of mice in vivo, when compared to the MC group. This additional evidence suggests that Glory LP16 is capable of restoring the CTX-induced immunocompromise observed in mice with low cellular immunity. The objective of this study was to investigate the impact of CTX-induced immunocompromise on cellular immunity in mice.

The hemolytic vacuole number and serum hemolysins analysis are key indicators for assessing the antibody secretion capacity and thus reflecting the immune status of the organism. The hemolytic vacuole count reflects the number of antibody-producing cells in the spleen, which can visually reflect the activity of the humoral immune response^33^. Furthermore, the serum hemolysin content can be used to reflect the status of humoral immunity. The experimental results demonstrated that, in comparison to the MC group, Glory LP16 was capable of restoring the number of hemolytic vacuoles and serum hemolysin levels, thereby providing further evidence that Glory LP16 is able to enhance the humoral immune capacity of mice.

NK cell activity and phagocytosis are often used to comprehensively assess the body’s non-specific immunity^34^. NK cells, as an important part of the human immune system, play a great role in antiviral and immunomodulation. Macrophages, as a key class of immune cells, have an important role in the clearance of foreign and damaged cells. The correlation between the rate of clearance of carbon particles in vivo and the concentration of carbon in the blood is commonly used to assess the functional status of macrophages. Bu YS gavage of Lactobacillus plantarum YRL4 significantly increased phagocytic activity and gene expression of IL-1β and TNF-α in mouse peritoneal macrophages, which is beneficial for enhancing the immune system under immunosuppression^29^. You YJ using Lactobacillus rhamnosus LM 1019 treatment increased the number of T-lymphocytes (including NK cells) in the spleen, indicating its potential to enhance the immune cell population^35^. The results showed that *Lacticaseibacillus paracasei* Glory LP16 was able to increase NK cell activity, NK cell activity, carbon profile/phagocytic index α, macrophage phagocytosis, and phagocytic index in mice, suggesting that Glory LP16 can modulate non-specific immunity in mice.

The gut is the body’s largest immune organ^36^. The gut not only absorbs the necessary nutrients for the body to maintain normal life activities, but also needs to resist the invasion of pathogens^37^. Colon tissue sections showed that different concentrations of Glory LP16 could alleviate intestinal barrier damage caused by CTX in immunocompromised mice.

A large number of studies have shown that intestinal flora participates in various metabolisms of the human body, and regulate the intestinal mucosal immunity of the human body, which in turn exerts the immunomodulatory effects of the human body^38^. Cyclophosphamide induces intestinal flora disorders in immunosuppressed mouse models^16^. Adding a certain dose of *Lacticaseibacillus paracasei* Glory LP16 can significantly increase the Chao1 index of immunosuppressed mice, change the Alpha index of the flora, improve the richness and diversity of the intestinal flora, and restore the intestinal flora disorder in immunosuppressed mice. The HP group is closer to the flora structure of the NC group This is consistent with previous reports that probiotics can modulate immunosuppression. The microbial communities of the gut contents of mice were evaluated at the phylum and genus levels. At the phylum level, Glory LP16 intervention resulted in a decrease in the relative abundance of the anabolic phylum and an increase in the abundance of the thick-walled phylum in mice, with F/B ratios closer to those of the NC group. *Lactobacillus* and other bacteria in the thick-walled phylum can hydrolyse carbohydrate substances to produce short-chain fatty acids such as butyric acid, reflecting the significant changes in intestinal flora, Glory LP16 can restore the disruption of intestinal flora, the abundance of the thick-walled phylum was reduced by Liu cyclophosphamide treatment^38^, and the abundance of the thick-walled phylum was increased by Zeng ZB. treatment^39^. At the genus level, the relative abundance of *Helicobacter*, *Mucispirillum*, *Oscillibacter* and *Enterorhabdus* was abnormally elevated after Glory LP16 intervention. The relative abundance of *Bifidobacterium*, *Lactobacillus*, *Alloprevotella*, and *Lachnospiraceae NK4A136 groups* was significantly increased. *Bifidobacteria* can promote the production of acetic acid in the body, and the increase of *Bifidobacteria* and *Lactobacillus* can promote the increase of beneficial bacteria in the body, thus improving the intestinal barrier and inhibiting the increase of harmful intestinal bacteria^40^. These results indicate that *Lacticaseibacillus paracasei* Glory LP16 can effectively improve the imbalance of intestinal flora in immunosuppressed mice and restore them to normal level.

Short-chain fatty acids (SCFAs) not only provide energy for the body, but also participate in lipid metabolism in the body and protect the intestinal mucosal layer barrier. Meanwhile, butyrate in SCFAs can promote the differentiation of T cells^42^. Short-chain fatty acids can promote the protection of the intestinal mucosal layer, thus playing a key role in the body’s immune barrier. The results showed that compared to the model group, supplementation with Glory LP16 increased the levels of acetic, propionic, and butyric acids, with butyric acid levels rising close to those of the normal group. Butyrate was found to alleviate pathological abnormalities in cecum tissue by Nozu^43^. It was shown that *Lacticaseibacillus paracasei* Glory LP16 can regulate the intestinal short-chain fatty acid (acetic, propionic and butyric acid) content to improve the body’s immunity^44^.

## Conclusions

The results showed that *Lacticaseibacillus paracasei* LP16 could significantly improve the body weight, thymus index and spleen index of immunocompromised mice. It can regulate the cellular immunity, humoral immunity and non-specific immunity of mice. The colonic morphology of mice tended to normalize; Regulate the imbalance of intestinal flora and increase the content of short-chain fatty acids in the intestine of mice. In conclusion, *Lacticaseibacillus paracasei* Glory LP16 can enhance immune function, and the effect of Glory LP16 high-dose group (1.6 × 10^8^ CFU/ mouse) is better, which provides a theoretical basis for the application of *Lacticaseibacillus paracasei* Glory LP16 in probiotic microecological products.

## Methods

### Materials and instruments

*Lacticaseibacillus paracasei* Glory LP16 powder, Jinhua Galaxy Biotechnology Co., Ltd. The isolation and identification were taken as *Lacticaseibacillus paracasei* Glory LP16; SPF-grade male BALB/c mice, 6–8 weeks old, weighing 18–22 g, provided by Liaoning Changsheng Biotechnology Co., Ltd, Animal Licence No.: SCXK (Liao) 2020–0001. Animal experiments were reported as described in the ARRIVE guidelines. The experimental animals were kept in a barrier environment animal room with a temperature of 20–23℃, and the light and dark alternated for 12 h, during which they could drink and eat freely. Sheep red blood cells (SRBC), Yuhuan Southern Reagent Co., Ltd; cell culture medium, GIBCO, USA; foetal bovine serum (FBS), Gibco, USA; Concanavalin A (Con A), Sigma, USA; Drabkin’s solution, Sigma, USA; NK-1.1-PE (108,707), Anolon (Beijing) Biotechnology Co.

### Experimental methods

#### Animal experimentation

BALB/c mice were randomly divided into 5 groups of 12 mice each. The control group and the model group were irrigated with saline for 30 d (Table 3). The low, medium and high (1.6 × 10^6^ CFU/mouse, 1.6 × 10^7^ CFU/mouse, 1.6 × 10^8^ CFU/mouse) dose groups were infused with Glory LP16 for 30d. On the 1st-3rd d, except for the control group which was injected with saline intraperitoneally, the remaining mice in the groups were injected intraperitoneally with 100μL of CTX (40 mg/kg), respectively. An immunocompromised mouse model was established. 12 h after the last dose, the weight of mice was recorded and the cervical vertebrae were dislocated, and killed, and the thymus and spleen were aseptically extracted to record the weight.

**Table 3:** Grouping of animal experiments.

Ordinal number	Groups	Number of animals	Quantity of feed	Gavage measurements (in terms of daily intake of animals)
1	Negative Control(NC)	12	Free-feeding	Same volume of saline
2	Model Control(MC)	12	Free-feeding	Subcutaneous injection of cyclophosphamide
3	Low-dose Control(LP)	12	Free-feeding	Subcutaneous injection of cyclophosphamide + Glory LP16(1.6 × 10^6^ CFU)
4	Glory LP16 Medium-dose Control(MP)	12	Free-feeding	Subcutaneous injection of cyclophosphamide + Glory LP16(1.6 × 10^7^ CFU)
5	Glory LP16 High-dose Control(HP)	12	Free-feeding	Subcutaneous injection of Cyclophosphamide + Glory LP16(1.6 × 10^8^ CFU)

Different lowercase letters indicate significant differences (*p* < 0.05), n = 12.

#### Measurement of indicators of immune function

The body mass, immune organ index, cellular immunity, humoral immunity, monocyte-macrophage function and NK cell activity in non-specific immunity were measured with reference to the "Technical Code for the Inspection and Evaluation of Health Foods" (2023 edition)^20^.

#### Body mass and immune organ index

Body weight was recorded 12 h after the last feeding. After cervical dislocation, the nucleus and spleen were aseptically removed, the remaining blood was dried with filter paper, weighed, divided by the rat’s body weight (g) and multiplied by 10 to obtain the nucleus and spleen indices, respectively.

#### Measurement of indicators of cellular immunity

##### Con A induced mouse splenic lymphocyte assay absorbance

Spleen was taken, sieved through 200 mesh sieves, washed twice with Hank’s solution, and centrifuged for 10 min each time (1000 r/min) (SORVALL ST8/8R, TOMOS, USA;). The cell concentration was adjusted to 3 × 10^6^ cells/mL, and added into 24-well culture plate, 1 mL per well, one well was added with 75 μL of Con A liquid, and the other well was used as the control, and placed in 5% CO_2_, 37 ℃ CO_2_ incubator for 68 h. The supernatant of each well was aspirated off 0.7 mL, and 0.7 mL of calf-serum-free RPMI1640 culture medium was added, and at the same time, MTT (5 mg/mL) was added 50 μL/well. After 4 h, add 1 mL of acid isopropyl alcohol to each well, loaded into 96-well culture plates, and using an enzyme marker, OD_570 nm_ was measured (SMATBCD, BIOTEK, USA).

##### Delayed-type hypersensitivity

Mice were injected intraperitoneally with 0.2 mL of 2% (v/v) SRBC. After 4d, the thickness of the metatarsal region of the left hind foot was measured, and 20 μL of 20% (v/v) SRBC was injected subcutaneously at the measurement site, and the same location was measured 24 h later.

#### Measurement of humoral immunity indicators

##### Antibody producing cell assay

Surface medium 45–50 ℃ water bath, mixed with an equal amount of pH 7.2–7.4, 2 times the concentration of Hank’s solution, divided into each tube 0.5 mL, add 50μL 10% SRBC, 20μL splenocyte suspension, to make parallel slices, placed in a carbon dioxide incubator for 1–1.5 h, add SA buffer diluted with complement, warmed for 1–1.5 h, and counted the number of hemolyzed vacuoles.

##### Serum hemolysins measurement

Mice were injected intraperitoneally with 0.2 mL of 2% (v/v) cell suspension. After 5d, blood was taken from the eyeballs and centrifuged for 10 min at 2000 r/min for 1 h afterward, and serum was collected. Sample wells: 50μL of SA buffer was diluted; blank control wells: 50 μL of SA buffer, 25 μL of 10% (v/v) SRBC, and 50 μL of complement were added, and centrifugation was performed horizontally at 1500 r/min for 10 min at 37 ℃ for 30 min in a constant temperature incubator. Each supernatant was taken 50 μL and added to the 96-well culture plate, and Drabkin’s solution was added 150 μL. Half of the hemolyzed wells were set up and the serum was added. For half of the hemolyzed wells, add 12.5 μL of 10% (v/v) SRBC, and then add Drabkin’s solution to 200 μL. Mix well, leave for 10 min, and measure the OD_540 nm_.

1 $${\text{HC}}_{50} = {\text{dilution}}\;{\text{factor}} \times \frac{{{\text{Sample}}\;{\text{optical}}\;{\text{density}}\;{\text{value}}}}{{{\text{Optical }}\;{\text{density}}\;{\text{at}}\;{\text{half}}\;{\text{of}}\;{\text{SRBC}}\;{\text{haemolysis}}}}$$

#### Measurement of non-specific immunological indicators

##### Mouse carbon profile assay

Mice were injected with India ink (10 mL/kg) in the tail vein. At 2 or 10 min after injection, 20 μL of blood was taken and immediately added to 2 mL of 0.1% Na_2_CO_3_ solution. OD_600 nm_ was measured, and Na_2_CO_3_ solution was used as a blank control. The phagocytic index α was calculated according to the following formula:

2 $$K = \frac{{{\text{lgOD1}} - {\text{lgOD2}}}}{{{\text{t2}} - {\text{t1}}}}$$

Mice were executed, livers and spleens were removed, dirty blood stains were drained by filter paper and weighed separately. The phagocytic index was used to express the ability of carbon contouring in mice. The phagocytic index α was calculated according to the following formula:

3 $${\text{phagocytosis}}\;{\text{index}}\;\alpha = \frac{{{\text{weight}}}}{{{\text{liver }}\;{\text{weight + splenic}}\;{\text{weight}}}} \times \sqrt[3]{K}$$

##### Phagocytosis of chicken erythrocytes by abdominal macrophages in mice

After preparing 20% (v/v) chicken red cell suspension, each mouse was intraperitoneally injected with 1 mL of 20% chicken red cell suspension. After 30 min, the mice were executed by cervical dislocation. Mice were injected into the peritoneal cavity with 2 mL of saline. A 1 ml drop of the peritoneal wash was aspirated onto a microscope slide and incubated in an incubator at 37 °C for 30 min. The incubated lotion was fixed with 1:1 acetone-methanol solution and stained with 4% (v/v) Giemsa-phosphate buffer for 3 min. Macrophages were counted under the oil mirror, 100 macrophages were counted per tablet, and phagocytosis rate and phagocytosis index were calculated as follows:

4 $$Engulfment \;percentage \% = \frac{{{\text{Number }}\;{\text{of }}\;{\text{macrophages}}\;{\text{phagocytising}}\;{\text{chicken}}\;{\text{erythrocytesuu}}}}{{{\text{Number }}\;{\text{of }}\;{\text{macrophages}}\;{\text{counted}}}} \times 100$$

5 $${\text{phagocytosis }}\;{\text{index}} = \frac{{{\text{Total}}\;{\text{number}}\;{\text{of}}\;{\text{phagocytosed}}\;{\text{chicken}}\;{\text{erythrocytes}}}}{{{\text{Number }}\;{\text{of}}\;{\text{macrophages}}\;{\text{counted}}}}$$

##### NK cell activity assay

Natural release wells:100 μL of target cells, 100 μL of culture medium, placed in 96-well U-plates; maximum release wells: 100 μL of target cells, 100% NP40 100 μL. The 96-well plates were cultured at 37℃ and 5%CO_2_ for 4 h, and then centrifuged at 1500r/min for 15 min. One hundred μL each of supernatant and LDH base solution was added to each well, and after 5 min, thirty μL of 1 mol/L HCl was added to each well in a 96-well plate, and the OD_490 nm_ value was determined. NK cell activity was calculated according to the following formula:

6 $${\text{NK}} - {\text{cell }}\;{\text{activity}}\;{ }\% = \frac{{{\text{OD }}\;{\text{of}}\;{\text{ reaction}}\;{\text{hole}} - {\text{OD }}\;{\text{of}}\;{\text{natural}}\;{\text{release}}\;{\text{hole }}}}{{{\text{OD }}\;{\text{of}}\;{\text{maximum }}\;{\text{release }}\;{\text{hole}} - {\text{OD }}\;{\text{of}}\;{\text{natural}}\;{\text{release}}\;{\text{hole }}}} \times 100\%$$

#### Histopathological observation of the colon

Mouse colon tissues were fixed with 4% (volume fraction) formaldehyde solution, then embedded sections with paraffin, deparaffinized with xylene and stained with hematoxylin–eosin (HE), and the histopathological changes of the mouse colon were observed under a light microscope with 200× magnification.

#### Analysis of the gut microbiome

After the mice were sacrificed, the intestinal contents of the mice were collected and stored in sterile tubes. The DNA was extracted from the intestinal contents of the mice by E.Z.N.A.® Stool DNA kit, and the purity and concentration of the DNA were detected by 1% agarose gel electrophoresis. The universal primers F:5 '-TAC GG R AGG C AG C Ag-3' and R:5 '-AGGTATCTAATCCT-3' were used to detect the 16SrRNav3-V4 region of gut microbiota reaction, PCR) amplification. PCR products were purified by AM Pure XT Beads and quantified by Qubit. The purified PCR products were subjected to Paired-End sequencing on Illumina platform. QIIME (V 1.9.1) software was used to filter raw data to obtain high-quality sequences. These high-quality sequences were clustered into operational taxonomic units (OTUs) with ≥ 97% similarity using the UPARSE tool. Based on the Green genes database, the taxonomic information of OTUs was annotated at the phylum and genus levels using the Py NAST software.

#### Quantitative analysis of short-chain fatty acids

The mouse intestinal contents (0.80 g) were collected, dissolved in sterile PBS, and prepared into a 10% suspension. Five hundred μL suspension was mixed with 100μL of crotonic acid metaphoric acid solution, frozen at −30℃ for 24 h, and centrifuged at 8000 r/min for 3 min to remove proteins and other impurities. The supernatant was filtered through a 0.22 μm stream filter, and the SCFAs (acetic acid, propionic acid, and butyric acid) content was determined by gas chromatography. The chromatographic conditions were as follows: the column temperature was 75 ℃, then increased to 180 ℃ at 20 ℃/min for 1 min, and then increased to 220 ℃ at 50 ℃/min for 1 min. The injection temperature was 250 ℃, the injection volume was 1.0 μL, and the split ratio was 5∶1. The carrier gas was high purity nitrogen. The flow rate was 2.5 mL/min for 6.5 min, and then increased to 2.8 mL/min for 2 min. Hydrogen flame ionization detector; Temperature 250 ℃; The tail blow rate was 20 mL/min.

### Statistics and analyses

IBM SPSS Statistics 23 statistical software was applied to analyze one-way ANOVA and between-group LSD test for the measured data that conformed to normal distribution. The experimental data were expressed as mean ± standard deviation (x ± S), one-way ANOVA method for data processing and LSD method for two-by-two comparisons, with a significant difference of (*p* < 0.05).

## Data Availability

All data generated or analysed during this study are included in this published article.
